# Coreceptor and Cytokine Concentrations May Not Explain Differences in Disease Progression Observed in HIV-1 Clade A and D Infected Ugandans

**DOI:** 10.1371/journal.pone.0019902

**Published:** 2011-05-31

**Authors:** Edward Wright, Susan Mugaba, Paul Grant, Rosalind Parkes-Ratanshi, Lieve Van der Paal, Heiner Grosskurth, Pontiano Kaleebu

**Affiliations:** 1 MRC/UVRI Uganda Research Unit on AIDS, Uganda Virus Research Institute (UVRI), Entebbe, Uganda; 2 Division of Infection and Immunity, University College London, London, United Kingdom; 3 Department of Virology, University College London Hospital, London, United Kingdom; 4 London School of Hygiene and Tropical Medicine, London, United Kingdom; Karolinska Institutet, Sweden

## Abstract

**Background:**

The use of cellular coreceptors and modulation of cytokine concentrations by HIV to establish a productive infection is well documented. However, it is unknown whether the expression of these proteins affects the course of HIV clade A and D disease, reported to have different progression rates.

**Methodology/Principal Findings:**

We investigated whether the number of CD4^+^ T-cells expressing CCR5 or CXCR4, the density of these coreceptors and concentrations of specific immune proteins linked to HIV pathogenesis vary between individuals infected with HIV clade A or D. We undertook additional analyses stratifying participants by early (CD4>500 cells/µl) or late (CD4<200 cells/µl) disease stage. Whole blood samples were taken from 50 HIV-1 infected individuals drawn from cohorts in rural south-west Uganda. Late stage participants had less than half the number of CD4^+^/CCR5^+^ T-cells (p = 0.0113) and 5.6 times fewer CD4^+^/CXCR4^+^ cells (p<0.0001) than early stage participants. There was also a statistically significant difference in the density of CXCR4 on CD4^+^ cells between clade A and D infected early stage participants (142 [A] vs 84 [D]; p = 0.0146). Across all participants we observed significantly higher concentration of Th_1_ cytokines compared to Th_2_ (66.4 vs 23.8 pg/ml; p<0.0001). Plasma concentrations of IFNγ and IL-2 were 1.8 and 2.4 fold lower respectively in Late-D infected participants compared to Late-A participants. MIP-1β levels also decreased from 118.0 pg/ml to 47.1 pg/ml (p = 0.0396) as HIV disease progressed.

**Conclusions/Significance:**

We observed specific alterations in the abundance of CD4^+^/CCR5^+^ and CD4^+^/CXCR4^+^ T-cells, and concentrations of immune proteins across different HIV clades and as infection progresses. Our results suggest that these changes are unlikely to explain the observed differences in disease progression between subtype A and D infections. However, our observations further the understanding of the natural progression of non-clade B HIV infection and how the virus adapts to exploit the host environment.

## Introduction

Based on the World Health Organisation figures from 2009, human immunodeficiency virus (HIV) is responsible for 1.9 million deaths and currently infects over 33 million people worldwide. Isolates of this retrovirus can be split into M, N, O and a newly identified P group based on phylogenetic analysis [1]. The main group (M) accounts for more than 90% of infections, while isolates from the N group, limited to roughly 10 isolates, and the Outlier group (O) have been detected in Cameroon. P group isolates are closely related to gorilla retroviruses. Within the M group there are over nine different clades or subtypes, circulating recombinant forms and unique recombinants, with distinct geographical distributions. Clade B is predominant in Europe and North America, with clade C the most common in Africa and Asia.

There are therapeutic antivirals available that target different stages of the virus lifecycle. While these are able to control viral replication the virus is not completely cleared and there is no efficacious prophylactic vaccine [2]. HIV is therefore a major burden on many countries but particularly those that are resource-limited as they also suffer from higher prevalences and incidence rates. The natural course of the infection can be split into three stages [3]: (1) Acute phase - lasting several weeks, during which a spike in viral replication occurs before the immune system is able to control this. (2) Clinical latency - when the host immune response is able to limit virus replication. This is usually maintained for 4–8 years, although it can be greater in long term non-progressors/elite viral supressors (3) The final stage – during which the CD4^+^ T-cells in which the virus replicates are depleted. The latter is referred to as acquired immunodeficiency syndrome (AIDS) and multiple co-infections can occur that cause the death of the infected individual unless effective therapy is employed.

Attachment of the virus is mediated through the binding of the viral envelope gp120 protein to the cellular CD4 receptor. This results in a conformational change in the virus protein allowing another envelope protein (gp41) to interact with the CCR5 or CXCR4 cellular co-receptor facilitating fusion of the cell membrane and virus envelope and consequently virus entry. The importance of chemokine receptors CCR5 and CXCR4 in HIV-1 infection was first reported in 1995/6 [4], [5]. Since then much work has been undertaken to characterise the interaction between HIV-1 and the coreceptors it uses in order to further our understanding of HIV replication and pathogenesis (reviewed elsewhere [6]). As mentioned, the early stages of HIV infection are mainly characterised by a high CD4 count (>500 cells/µl) and the majority of circulating viruses are CCR5-tropic (R5). These exhibit slow/low replication kinetics, are non-syncytium inducing and predominantly infect activated CD4^+^ cells and macrophages [7], [8]. In contrast, at the late stage of disease progression (CD4 count less than 200 cells/µl) CXCR4 using virus isolates (X4) are abundant, mainly in clade B infected patients, and exhibit rapid replication kinetics. They are characterised by the ability to form syncytia and infect memory and naïve CD4^+^ cells [9]. The fact that these slow and rapid phenotypes are predominantly observed at early and late stages of HIV disease respectively can partly be explained by the fact that R5 HIV-1 accounts for the vast majority of infections [6], although possible cases of X4 HIV-1 transmission have been reported [10].

Various immunological and viral factors may affect coreceptor expression and consequently influence how quickly HIV positive individuals progress to AIDS. For example, specific HLA alleles have been shown to influence the potency of anti-Gag T-cell responses [11] and the actual switch in coreceptor usage, or phenotypic switch, from R5 to X4, is associated with faster CD4 decline and consequently a more rapid HIV disease progression [12], [13], [14]. However, as X4 isolates are more sensitive to neutralising antibodies, it could be that they are “opportunistic infections” appearing at late stages of HIV disease when the humoral host immune response has been greatly exhausted. It should also be noted that while this switch has primarily been reported in clade B [13] it has also occurred in clades C [15], [16], A and D [17], [18] to varying degrees and some viral isolates are able to use both CCR5 and CXCR4 [19] or alternative coreceptors [20], [21] *in vitro* (also reviewed elsewhere [7]).

In HIV-1 positive cohorts of African adults, individuals infected with clade D progressed faster to AIDS than those with clade A [22], [23], [24]. This difference in disease progression could be due to clade D viruses switching from R5 to X4 phenotype earlier in infection, at higher CD4^+^ cell counts, than clade A isolates [25]. However, cellular factors such as interleukin-7 (IL-7), IL-4, tumour necrosis factor α (TNFα), RANTES and interferon γ (IFNγ), as well as the HIV-1 accessory protein Nef, have all been shown to alter the level of chemokine receptor surface expression. Therefore, they may also influence the rate of disease progression [26], [27], [28], [29]. Similarly, it is possible that a select group of immune proteins, so called anti-HIV cytokines and chemokines known to influence the risk of HIV-1 infection and consequent disease progression [30], could play a role in the more rapid disease progression observed in clade D infection.

Here we report the findings of a cross-sectional study to investigate whether there are differences in the quantity of CD4^+^ T-cells expressing CCR5 or CXCR4, the cell surface density of these receptors and the concentrations of certain anti-HIV-1 cytokines and chemokines between clade A or D infected participants. We also investigate the levels of these factors in individuals at early (CD4>500 cells/µl) or late (CD4<200 cells/µl) stages of HIV-1 disease.

## Methods

### Objectives

To determine if the abundance of HIV-1 coreceptors and anti-HIV cytokines/chemokines differs between clade A and D HIV-1 infected individuals. To compare the same variables at early and late stages of HIV-1 disease.

### Participants

Whole blood samples were taken from 50 highly active antiretroviral therapy (HAART) naïve individuals. Twenty-five individuals had a CD4 count of less than 200 cells/µl and 25 a CD4 count of more than 500 cells/µl. The participants were drawn from two clinical research cohorts, both of which were established by the MRC/UVRI Uganda Research Unit on AIDS in a rural area of south-west Uganda. Thirty seven individuals (none of whom were receiving HAART at the time of this study) were selected from the Rural Clinical Cohort (RCC) in Masaka district [31]. Of these individuals 25 had a CD4 count >500 cells/µl and 12 had a CD4 count of <200 cells/µl. As individuals in the RCC are offered HAART, there were few with a CD4^+^ T-cell count below 200 cells/µl who were still HAART naïve so we were not able to recruit all participants from the RCC. Therefore, the remaining 13 individuals with low CD4 counts were selected from a cohort recruited to investigate an innovative intervention against cryptococcal disease also based in Masaka district [32]. None of the participants had current opportunistic infections. To enable a comparison of immune markers between HIV negative and positive individuals, four HIV negative participants were randomly selected from the RCC.

### HIV coreceptor staining

Blood was collected in EDTA vacutainers (Becton Dickinson [BD], UK) and transported to the laboratory where it was either used immediately or plasma isolated and stored. For each sample, 100 µl aliquots of fresh, whole blood were stained with CD3-FITC, CD4-PerCP-Cy5.5, and either CCR5-PE, CXCR4-PE or their respective isotype controls (BD PharMingen [BDP], UK) to determine the percent of CD4^+^ cells expressing CCR5 or CXCR4. A FACScan and CellQuest Pro (BD, UK) were used to acquire 3x10^5^ events for each condition. Each sample was gated on the CD3^+^/CD4^+^ population before any analysis was undertaken. The relative size (forward scatter^2^) of the CD4^+^ T-cells for each individual was recorded along with the mean fluorescent intensity (MFI) of CCR5 and CXCR4. We used these values to calculate the density of CCR5 and CXCR4 on the selected cell population, one possible method for determining relative receptor densities.

### Cytometric bead array (CBA)

A human cytokine/chemokine CBA flex set kit (IFNγ, TNFα, IL-2, IL-10, IL-5, IL-4, MIP-1α, MIP-1β and RANTES) was used as described by the manufacturer (BD, UK) with 50 µl of cryopreserved plasma from each participant. CBA Analysis Software (BD, UK) was used to analyse the data generated. The standards for this assay cover 10–5,000pg/ml, however the standard curve defines the outer limits of detection. The theoretical limit of detection is defined as the corresponding concentration at two standard deviations above the median fluorescence of the negative control (0 pg/ml) using a 4- or 5-parameter curve. Therefore, it is possible to determine values for samples that fall within the linear range of the curve, 0–15,000 pg/ml for the dilution series we used in this study.

### HIV testing, subtyping and viral load

HIV serostatus was confirmed using two commercial ELISA kits (Wellcozyme Recombinant HIV EIA (Murex Biotech, UK) and Recombigen HIV-1/HIV-2 [Trinity Biotech, UK]) and a western blot kit (New Lav Blot 1 [Bio-Rad, France]). CD4^+^ T-cell counts were determined using the BD FACScount system (BD, UK). HIV viral load testing and genetic subtyping was undertaken using qRT-PCR and direct sequencing of the reverse transcriptase gene [33]. For 5 participants on whose specimens sequencing was not successful, heteroduplex mobility assays (HMA) were undertaken [34].

### Statistical methods

To determine whether variables were normally distributed D'Agostino & Pearson omnibus normality tests were performed. If all or the majority of data in each set was normally distributed mean values were used, otherwise median values were cited. Analyses undertaken were pairwise comparisons rather than factorial analyses involving the four groups. Therefore, corresponding two-sample t-tests (for variables which could be regarded as being normally distributed) or Mann-Whitney tests (for variables for which we could not assume normality) were performed to examine whether the selected variables differed between the two groups. All reported p-values are two-sided using a level of significance of 0.05. Statistical analyses were conducted using GraphPad Prism (GraphPad Software, San Diego, USA).

### Ethical considerations

Written informed consent was obtained from all participants and ethical approval for the study was given by the ethics committee of the Uganda Virus Research Institute and the Uganda National Council for Science and Technology. HAART was provided to all eligible participants shortly after specimen collection for this study.

## Results

**Figure 1:**
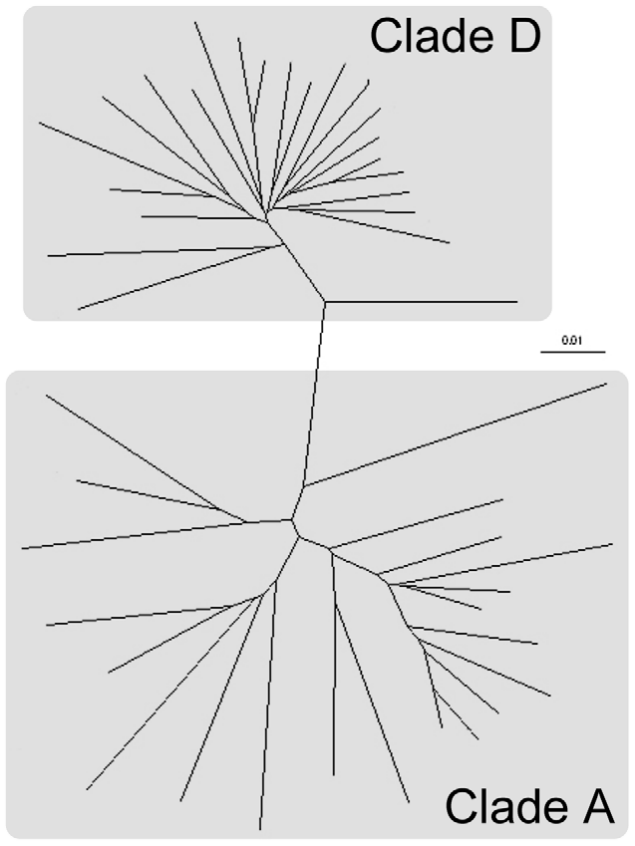
Radial tree showing degree of identity between the study participants' HIV isolates. RT-PCR was undertaken to amplify sequence from the reverse transcripase gene that was subsequently used to construct the tree. The branch lengths and scale correspond to the number of substitutions/mutations per site.

For analyses participants were placed into one of fours groups according to disease stage and HIV subtype: Early-A, Early-D, Late-A and Late-D. Of the 50 participants enrolled in this study clades A and D were the only subtypes present (25 participants each; Figure 1). Sixty percent of early stage participants were infected with clade A while this was reversed in the late stage group with 60% infected with clade D (Table 1). The mean CD4^+^ count for early stage individuals was 736 cells/µl (95% confidence interval [C.I.] 663–808) compared to 146 cells/µl (95% C.I. 122–169) for the late stage group. The CD4^+^ counts were similar for A and D infected participants at both time points (Table 1). As expected the mean CD4^+^ count in HIV negative participants was much higher (950 cells/µl [95% C.I. 513–1388]; n = 4). There was a similar age distribution between the four groups, with the Early-D group having the highest (40.3 [32.1–48.4]; mean age [95% C.I.] in years). There were more females than males in the late group (64% vs 36%) but this was skewed by the Late-A group comprising 90% females, a chance result due to the sampling strategy used. As expected, individuals in the late group were also at more advanced stages of HIV disease according to WHO staging compared to early individuals (Early  =  2.2 [1.8–2.6], Late  =  2.8 [2.5–3.1]; mean stage [95% C.I.] in years). The infecting subtype did not affect WHO disease staging. There was a statically significant difference in HIV viral load measurements between participants in early versus late time points (early: 14,021 [4058–23983]; mean viral load [95% C.I.]. Late: (132,203 [44,944–219462]; mean viral load [95% C.I.]. p = 0.0126; unpaired t-test). While there was a trend for the Early-D and Late-D groups to have higher viral load measurements than the corresponding clade A infected groups, this difference was not significant (Table 1).

**Table 1:** Characteristics of study participants.

	HIV disease stage				Total (n = 50)^a^
	Early (CD4>500)		Late (CD4<200)		
	Clade A^a^	Clade D	Clade A	Clade D	
Number of participants	15	10	10	15	50
*Drawn from the RCC * ^b^	*15*	*10*	*5*	*7*	*37*
*Drawn from the CTR * ^b^	*0*	*0*	*5*	*8*	*13*
Age (years; mean)	36.7	40.3	36.8	37.1	37.6
	[30.3-43.2]	[32.1-48.4]	[26.3-47.3]	[33.8-40.4]	[34.5-40.6]
Gender: F/M (%)	47/53	50/50	90/10	56/44	56/44
WHO HIV disease	***1***: 33; ***2***: 27	***1***: 20; ***2***: 30	***1***: 0; ***2***: 20	***1***: 13; ***2***: 20	***1***: 18; ***2***: 24
stage (%)	***3***: 33; ***4***: 7	***3***: 40; ***4***: 10	***3***: 60; ***4***: 20	***3***: 54; ***4***: 13	***3***: 46; ***4***: 12
Mean CD4^+^ T-cell	724	754	147	145	441
count (cell/µl)	[624-823]	[628-879]	[116-179]	[108-182]	[348-533]
Mean viral load a	8,142	20,487	85,126	108,687	59,273
(copies/ml)	[3,115-13,169]	[1,134-42,108]	[14,268-184,519]	[22,167-195,207]	[25,408-93,139]

a The data shown in this table were calculated using all HIV positive participants enrolled in this study (n = 50), apart from HIV envelope subtype data and viral load measurements, where samples to obtain these data were not available for five participants. For these samples, four from the early group and one the late group, existing HMA data was used to subtype.

b RCC  =  Rural Clinical Cohort; CTR  =  Cryptococcal Trial Recruitment. The 95% confidence intervals for age, CD4^+^ T-cell count and viral load are given in square brackets beneath the mean value for these variables.

**Figure 2:**
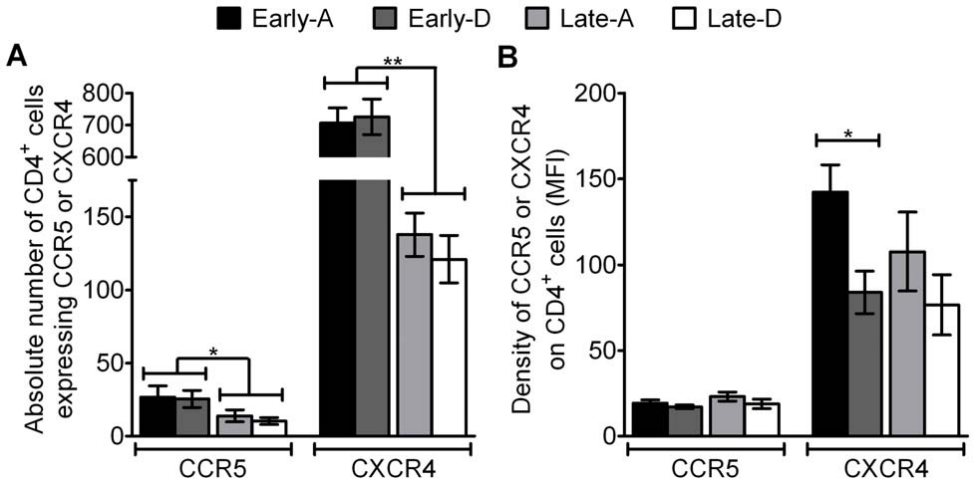
Coreceptor abundance stratified by HIV disease stage and clade. The mean absolute number of CD4^+^/CCR5^+^ and CD4^+^/CXCR4^+^ T-cells (**A**) and mean density of CCR5 and CXCR4 on CD4^+^ cells (**B**) are shown, with error bars representing standard error of the means. Results are shown for samples from participants at early (black and heavy shading bars) or late (light shading and white bars) stages of infection and for participants infected with HIV-1 clade A (black and light shading bars) or D (heaving shading or white bars). *  =  p<0.05 and **  =  p<0.0001, calculated using unpaired t-test.

We determined whether the abundance of HIV coreceptors, quantified by either the absolute number of total CD4^+^ T-cells expressing CCR5 or CXCR4, or their density on those cells, varied between clades or during the course of HIV infection. Prior to analysis each sample was gated on the CD3^+^/CD4^+^ cell population and statistical testing showed the majority of the coreceptor data obtained for this analysis was normally distributed (D'Agostino & Pearson omnibus normality test; data not shown). As a benchmark against which to compare results from HIV positive individuals, we determined the abundance of CCR5 and CXCR4 T-cells in four uninfected RCC participants. Absolute numbers of CD4^+^/CCR5^+^ and CD4^+^/CXCR4^+^ cells in HIV negative individuals were 99 cells/µl (55-143 95% C.I.) and 922 cells/µl (490-1354 95% C.I.) respectively. Analysing all HIV infected participants, there was 4.8 fold more CD4^+^ T-cells expressing CXCR4 (421 cells/µl [329-513]; mean [95% C.I.]) than CCR5 (19 cells/µl [13]–[25]; mean [95% C.I.]; p<0.0001, unpaired t-test). The mean number of CD4^+^/CCR5^+^ cells in participants at a late time point in HIV-1 infection was 2.2 fold lower compared to those at early stage groups, 12 and 26 respectively (p = 0.0113, unpaired t-test; Figure. 2A). The same trend was seen when comparing the mean number of CD4^+^ cells expressing the CXCR4 receptor in late vs. early stage groups. This was significantly higher in early stage HIV-1 infected participants than those at late time points (714 to 128 cells/µl; p<0.0001, unpaired t-test; Figure. 2A).

Similar to the difference observed in the absolute number of T-cells expressing the two coreceptors, the density of the CXCR4 (104.0 [85.06-122.9]; mean density [95% C.I.]) across all participants was 5.3 times higher than CCR5 (19.47 [17.15–21.79]; mean density [95% C.I.]; p<0.0001, unpaired t-test). These densities were also lower than mean densities observed in HIV negative participants (CCR5  =  27.2 and CXCR4  =  120). The density of the CCR5 coreceptor on the surface of CD4^+^ cells in HIV positive participants was similar between the four groups. However, the mean density of CXCR4 was 1.7 fold lower in the Early-D group (83.94 [55.84–112]; mean density [95% C.I.]) than the Early-A group (142.2 [108.6–176.3]; mean density [95% C.I.]; p = 0.0146; unpaired t-test; Figure 2B).

**Figure 3:**
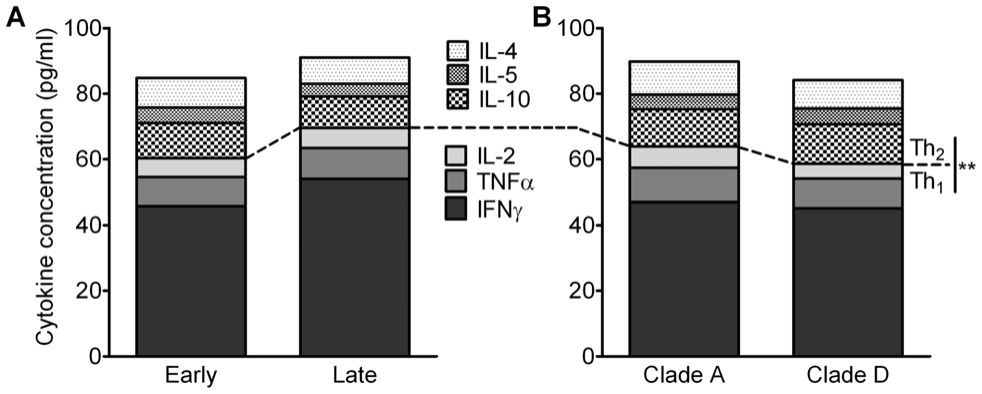
Total cytokine concentrations in study participants. Bar graphs represent cytokines concentrations (pg/ml) as a proportion of the total concentration of Th_1_ and Th_2_ cytokines measured. These have been stratified by HIV-1 disease stage (**A**) or HIV-1 clade (**B**) with shaded and patterned areas corresponding to Th_1_ and Th_2_ cytokines respectively. **  =  p<0.0001 (Mann Whitney test) for total Th_1_ vs Th_2_ cytokines when compared across all HIV positive study participants (n = 50).

As other cell surface molecules and cytokines are known to influence the clinical course of HIV-1 disease progression and the expression of HIV CCR5 and CXCR4 coreceptors, we measured the level of certain anti-HIV-1 cytokines (IFNγ, TNFα, IL-2, IL-10, IL-5 and IL-4) and chemokines (MIP-1α, MIP-1β, RANTES) in the plasma of each study participant. Most of the cytokine data was not normally distributed (D'Agostino & Pearson omnibus normality test; data not shown) so median values and corresponding significance tests were used for the analysis. These revealed that the combined concentrations of Th_1_ cytokines in all participants was significantly higher than Th_2_ concentrations (66.4 vs 23.9 pg/ml; p<0.0001, Mann Whitney test; Figure 3). The observed increase in Th_1_ cytokines (62.7 vs 71.0 pg/ml) and slight decrease in Th_2_ cytokines (24.6 vs 23.1 pg/ml) in individuals at the late stage of HIV disease when comparing with those at the early stage, were not significant (p = 0.4728 and p = 0.7269 respectively, Mann Whitney test; Figure 3A). There was a small decrease in the concentration of Th_1_ cytokines (58.6 [D] vs 63.9 [A] pg/ml) and similar levels of Th_2_ cytokines (25.5 [D] vs 25.9 [A] pg/ml) in clade D infected participants compared to clade A infected (Figure 3B). However, neither variation was significant.

**Figure 4:**
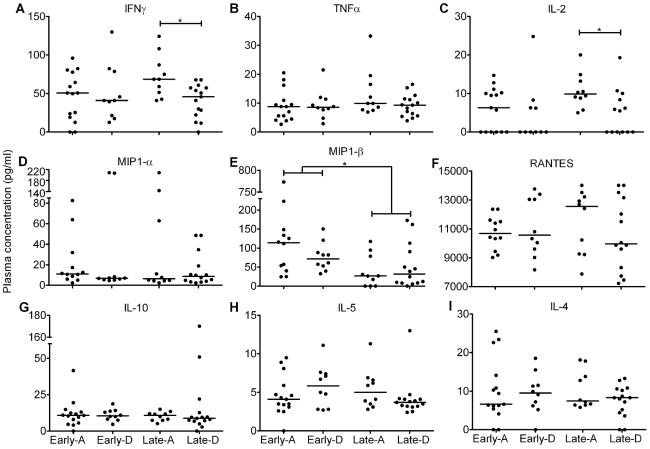
Individual profiles for all cytokines and chemokines measured. Vertical scatter plots show the median plasma concentration (horizontal black line; pg/ml) for the anti-HIV-1 cytokines (IFNγ, TNFα, IL-2, IL-10, IL-5 and IL-4) and chemokines (MIP-1α, MIP-1β and RANTES) measured as part of this study. Participants were analysed in four groups based on disease stage and HIV clade: Early-A, Early-D, Late-A and Late-D. *  =  p<0.05 and **  =  p<0.0001, calculated using Mann Whitney test.

Median concentrations of individual cytokines and chemokines were analysed across the four groups: Early-A, Early-D, Late-A and Late-D. These revealed comparable levels in the majority of participants. The range of median concentrations was 0–11.1 pg/ml (Figure 4) apart from IFNγ (50.70 pg/ml; Figure 4A), MIP-1β (53.39 pg/ml; Figure 4E) and RANTES (10611 pg/ml; Figure 4F). However, IFNγ levels in participants in the Late-D group were lower than those in Late-A (45.8 vs 68.7 pg/ml; p = 0.0065; Figure 4A). A similar pattern was observed when we measured IL-2 (0.0 vs 9.9 pg/ml; p = 0.0087; Figure 4C). Finally, there was a significant decrease in MIP-1β as the course of HIV disease progressed (81.9 vs 26.7 pg/ml; p = 0.0022; Figure 4E).

## Discussion

We have previously shown that HIV-1 clades A and D are predominant in our study population (42% and 58% respectively [35]). We observed a similar distribution within our current study participants, 50% for clade A and D. Analysis undertaken based on infecting HIV clade revealed that, with the exception of a lower density of CXCR4 on CD4^+^ T-cells for Early-D participants and lower concentrations of IFNγ and IL-2 for Late-D participants, levels of CCR5, CXCR4, cytokines and chemokines were comparable. These findings suggests that the level of coreceptor and cytokine/chemokine expression may not be the cause of the accelerated disease progression we previously reported in subtype D infection participants within our cohorts [23]. However, this could be caused by the phenotype switch occurring at an earlier disease stage [25] or a result of subtype D individuals having a higher viral load than subtype A individuals [36] (table 1). However, there could be other virological, cellular or immunological pressures influencing the above observations.

We observed a similar trend of marginally higher mean CD4^+^ counts in Ugandan women (483 [342–623 95% C.I.]) compared to Ugandan men (473 [345–601 95% C.I.]; p = 0.9197) in a previous study [37]. As neither of these trends was statistically significant it is unlikely that the high proportion of females in the Late-A group would lead to a bias in the other results reported in our present study. However, as expected the overall number of T-cells expressing the HIV coreceptors was higher in HIV negative participants than among HIV positive participants. Our data reveal that as infection progressed a concurrent depletion of the absolute number of CD4^+^/CCR5^+^ cells and CD4^+^/CXCR4^+^ cells occurred when comparing Early and Late stage groups. This seems counterintuitive to what we would expect if coreceptors were playing an active role in determining HIV phenotype. If the abundance of coreceptor expressing cells was driving the phenotypic switch we might observe higher levels of CD4^+^/CCR5^+^ cells in samples from individuals infected at early stages compared to those at late stages. Therefore, these variations are more likely to be a consequence of disease progression and not the cause of the coreceptor switch. This conclusion is consistent with a recent study by Poveda et al. who reported findings on the role of viral phenotype in pathogenesis of HIV-1 clade B [38]. That study also suggested that the phenotypic switch is a consequence rather than the cause of a decline in CD4 count.

The observed differences in numbers of CD4^+^/CCR5^+^ and CD4^+^/CXCR4^+^ cells between early and late disease stage groups in our study are consistent with those reported in other cohorts [39], [40]. However, those studies have not reported the density of CCR5 or CXCR4, or immune protein concentrations in HIV clade A and D infected individuals at early or late stages of HIV infection, all of which have been shown to influence HIV disease progression [30]. It is likely that the changes in the number of CCR5 and CXCR4 expressing CD4^+^ T-cells in our study were due to virus-mediated T-cell killing. This conclusion is also in agreement with the viral load data of the study participants. Individuals in the Late groups had lower numbers of CCR5^+^ and CXCR4^+^ T-cells which inversely correlated with the observed higher viral loads in these groups (CCR5 - r = −0.4 p = 0.0119; CXCR4 - r = −0.4 p = 0.0029; Spearman's Rank Correlation). However, HIV is known to cause immune activation, which can modulate the expression of CCR5 and CXCR4 [40], [41]. The high levels of CD4^+^/CXCR4^+^ cells and density of CXCR4 we found compared to CCR5 may also explain the greater pathogenicity of X4 HIV-1 observed in individuals infected with CXCR4 using HIV [42].

Various cytokines and chemokines have previously been implicated in influencing expression of CCR5 and CXCR4 on the surface of CD4^+^ cells [26], [27]. Because of this observation and taking the type 1/type 2 cytokine model of immune response to HIV-1 into account which predicts a switch in the balance of cytokines from Th_1_ to Th_2_ resulting in a more rapid disease progression, we were interested to know if the concentration of such immune proteins differed between the four groups of participants. Not only would this provide a better understanding of HIV disease progression, but it may also help to elucidate what triggers the phenotypic switch. Our data showed that the concentrations of a select group of cytokines/chemokines, when compared individually, differed only marginally between each group. However, larger and significant differences were apparent in the overall concentrations of Th_1_ and Th_2_ cytokines when all 50 participants were analysed. Th_1_ cytokines made up over 60% of the total concentration of cytokines measured. IFNγ accounted for the majority of the Th_1_ response and was primarily responsible for the increase in Th_1_ response as disease progressed. This is to be expected though as IFNγ is one of the main antiviral cytokines and viral load values were much higher in Late group participants. When analysing participants according to disease stage, the non-significant trend showing a decrease in Th_2_ and increase in Th_1_ cytokines is contrary to what we would predict but could be due to the fact that only a select group of immune proteins was measured. It is possible that other Th_2_ responses could be more substantially activated as the course of HIV infection progresses or that HIV primarily targets Th_1_ expressing cells at early times of infection so that this cell population becomes more rapidly depleted. In agreement with this possibility, analysis of cytokine/chemokine concentrations in our study failed to reveal a correlation with levels of CD4^+^/CCR5^+^ or CD4^+^/CXCR4^+^ cells, suggesting they are not directly driving the alternations in T-cell subsets (data not shown).

It is of interest to note that while we observed high median concentrations of IFNγ, MIP-1β and RANTES, there were low levels of IL-2, a cytokine that plays a key role in stimulating CD8^+^ T-cell responses. These differences could be caused by a number of environmental factors, such as opportunistic infections, time of sampling and age. However, we were able to minimise the possible influence of such factors through the study design, e.g. all participants underwent clinical examinations to exclude coinfections, blood samples were drawn at clinics held at the same time each day and the mean age and age range were similar across all groups. It is highly likely that the larger concentrations of IFNγ, MIP-1β and RANTES were due to their central role in controlling viral infections as they have previously been shown to potently inhibit HIV infection [43]. The level of these three predominant proteins did not correlate with CD4^+^ count or viral load (data not shown). Together these results suggest that the alterations we observed in certain cytokines and chemokine concentrations were either the result of a clade specific immune response or simply too small to be considered clinically relevant. However, it is noteworthy that the cytokine concentrations were within the same range as previously reported in another HIV positive Ugandan cohort [44]. Compared to the HIV negative Ugandans enrolled in that study, cytokine concentrations in our HIV positive participants were elevated, which could be explained by the highly activated status of their immune system due to HIV infection.

The changes observed in the abundance of CCR5 and CXCR4 chemokine receptors on CD4^+^ T-cells and of cytokines/chemokines during the natural course of non-clade B HIV-1 infection suggests that the phenotypic switch is not driven by changes in the levels of these receptors or anti-HIV cytokines. In addition, these observations are unlikely to explain the observed differences in disease progression between subtype A and D infected individuals. The factors responsible for triggering the switch in HIV coreceptor usage have yet to be conclusively identified. Our results further illustrate the complexity of the interaction between the virus and the host in HIV pathogenesis. Further work into the mechanism by which HIV manipulates the host environment as disease progresses may help tailor therapeutic interventions to each individual and also facilitate the development of new strategies to slow or halt the course of infection.
